# Dkk-3, a Secreted Wnt Antagonist, Suppresses Tumorigenic Potential and Pulmonary Metastasis in Osteosarcoma

**DOI:** 10.1155/2013/147541

**Published:** 2013-02-10

**Authors:** Carol H. Lin, Yi Guo, Samia Ghaffar, Peter McQueen, Jonathan Pourmorady, Alexander Christ, Kevin Rooney, Tao Ji, Ramez Eskander, Xiaolin Zi, Bang H. Hoang

**Affiliations:** ^1^Department of Oncology, CHOC Children's Hospital, 455 South Main Street, Orange, CA 92868, USA; ^2^Department of Orthopaedic Surgery, University of California, Irvine, 101 The City Drive South, Orange, CA 92868, USA; ^3^Department of Obstetrics and Gynecology, University of California, Irvine, 101 The City Drive South, Building 56, Suite 260, Orange, CA 92868, USA; ^4^Department of Urology, University of California, Irvine, 101 The City Drive South, Building 55, Suite 302, Orange, CA 92868, USA; ^5^Department of Pharmaceutical Sciences, University of California, Irvine, 101 The City Drive South, Orange, CA 92868, USA; ^6^Department of Orthopaedic Surgery and Chao Family Comprehensive Cancer Center, University of California, Irvine, 101 The City Drive South, Orange, CA 92868, USA

## Abstract

Osteosarcoma (OS) is the most common primary bone malignancy with a high propensity for local invasion and distant metastasis. Despite current multidisciplinary treatments, there has not been a drastic change in overall prognosis within the past 2 decades. Dickkopf-3 protein (Dkk-3/REIC) has been known to inhibit canonical Wnt/**β**-catenin pathway, and its expression has been shown to be downregulated in OS cell lines. Using *in vivo* and *in vitro* studies, we demonstrated that Dkk-3-transfected 143B cells inhibited tumorigenesis and metastasis in an orthotopic xenograft model of OS. Inoculation of Dkk-3-transfected 143B cell lines into nude mice showed significant decreased tumor growth and less metastatic pulmonary nodules (88.7%) compared to the control vector. *In vitro* experiments examining cellular motility and viability demonstrated less anchorage-independent growth and decreased cellular motility for Dkk-3-transfected 143B and SaOS2 cell lines compared to the control vector. Downstream expressions of Met, MAPK, ALK, and S1004A were also downregulated in Dkk-3-transfected SaOS2 cells, suggesting the ability of Dkk-3 to inhibit tumorigenic potential of OS. Together, these data suggest that Dkk-3 has a negative impact on the progression of osteosarcoma. Reexpressing Dkk-3 in Dkk-3-deficient OS tumors may prove to be of benefit as a preventive or therapeutic strategy.

## 1. Introduction

Osteosarcoma (OS) is the most common primary bone malignancy diagnosed in children and adolescents. With the current multidisciplinary treatments, 60–70% of patients with localized disease survive [1]. According to the Children's Oncology Group (COG) protocol for localized disease, standard therapy consists of neoadjuvant chemotherapy, including doxorubicin, cisplatin, and high-dose methotrexate, followed by surgical resection. After surgical intervention, adjuvant chemotherapy is given, dependent upon the degree of necrosis. Good responders to neoadjuvant therapy will show < 10% viable tumor and will be randomized to continue with adjuvant therapy. According to the European and American Osteosarcoma 1 Trial (EURAMOS 1), the five-year survival for good responders is 75–80% compared to poor responders who face survival percentages of 45–55%. Prognostic factors for OS include tumor site and size, primary metastases, response to chemotherapy and surgical remission [2, 3]. Osteosarcoma has a high tendency for local invasion and early metastasis. Unfortunately, with metastatic disease, the rate of 5-year overall survival is greatly reduced to 20–30%, and the 5-year event-free survival for patients with relapse is only 20% [4, 5]. Metastasis occurs primarily to the pulmonary fields. Even though there is no initial evidence of metastasis from baseline chest CT scans, it is thought that there are micrometastasis, creating further difficulties in treating this malignant process. Despite aggressive efforts to treat, the outcome of patients with OS has not significantly improved during the past two decades. This creates an opportunity for more effective targeted therapies.

The canonical Wnt/*β*-catenin signaling pathway has been shown to control multiple cellular processes, including cellular proliferation, cell fate determination, and differentiation in numerous developmental stages, from embryogenesis to adult tissue homeostasis [6–9]. Given the power of this central mediator, inhibition of Wnt/*β*-catenin signaling is a key potential strategy for cancer therapy. Our previous study on Dickkopf 3 (Dkk-3, also known as reduced expression in immortalized cells (REIC)) showed its ability to inhibit invasion and motility of OS cells via modulation of the Wnt-*β*-catenin pathway. We showed that Dkk-3 downregulates *β*-catenin nuclear translocation in osteosarcoma cells resulting in inhibition of downstream Wnt-mediated lymphoid enhancer factor/TCF activation [10]. 

The Dickkopf family comprises of four secretory proteins, Dkk-1, Dkk-2, Dkk-3, and Dkk-4. Human Dkk-1 inhibits Wnt signaling pathway (well known for its roles in embryogenesis and cancer) by binding to the transmembrane receptors (Lipoprotein receptor-related protein-5 and -6 (LRP5, LRP6) [11]. The secretory protein REIC/Dkk-3 mechanism of action for inhibiting Wnt signaling pathway is currently unknown, but its expression has been shown to be downregulated in various cancer cell lines, including prostate, renal, liver, pancreas, cervical, lung, melanoma, glioma, testicular, colon, and even osteosarcoma cancer cells [10–20]. 

 Our previous data showed that Dkk-3 inhibited *in vitro* invasion and motility of OS cell line SaOS2 by modulating the Wnt/*β*-Catenin pathway. Transfection of Dkk-3 and dominant-negative *LRP5 *into SaOS-2 cells significantly reduced the invasion capacity and motility [10]. In this study, we continued to focus on the effects of Dkk-3 *in vitro *and *in vivo *to assess its ability to decrease tumor progression in osteosarcoma and elucidated the potential molecular mechanism. 

## 2. Materials and Methods

### 2.1. Cell Lines, Compounds, Reagents, and Plasmids

Human OS cell lines 143B, 143.98.2, SaOS-2, MNNG-HOS, MG-63, and U2-OS (American Type Culture Collection), SaOS-LM7 (a gift from Dr. Eugenie Kleinerman, MD Anderson Cancer Center, Houston, TX, USA), and OS160 (a gift from Richard Gorlick, Albert Einstein College of Medicine, Bronx, NY, USA) were maintained in MEM-Alpha medium supplemented with 10% fetal bovine serum (FBS). Normal human osteoblasts (NHOSTs) were obtained from Cambrex Bio Science and maintained in Osteoblast Growth Media (Lonza). All cells were maintained in a 37°C incubator with humidified atmosphere of 5% CO_2_. *PcDNA3.1* Directional TOPO Expression vector was obtained from Invitrogen. Dkk-3 clone was constructed as previously described [10]. Antibodies against *β*-actin was from Santa Cruz Biotechnology, Inc. (Santa Cruz, CA, USA). Thymidine, 3-(4,5-dimethylthiazol-2-yl)-2,5-diphenyltetrazolium bromide (MTT) and propidium iodide were obtained from Sigma (Saint Louis, MO, USA). RNAzol B was purchased from Tel-Test (Friendswood, TX), and the Reverse Transcription System kit was from Applied Biosystems (Carlsbad, CA, USA).

### 2.2. MTT Assay

143B OS cells were plated at a density of 2 × 10^4^ cells per well in 24-well culture plates in 500 *μ*L of growth medium containing 10% FBS. After 24 hours, the cells were transfected with either PcDNA control or various colonies of Dkk-3. Following transfection, at 72 hours, 500 uL of MTT solution (final concentration of 1 mg/mL) was added to each well and incubated at 37°C for 1.5 hours. Once MTT solution was removed, 500 uL of dissolve buffer (4% 1 M HCl, 96% Isopropanol) was added, and plates were gently shaken for 5 minutes to dissolve the crystals. Cell viability was assessed by measuring the absorbance at 570 nm from a 96-well plate microplate reader (Bio-Rad, Hercules, CA, USA). Dose response curves for cell viability were generated as a percentage of vehicle-treated control.

### 2.3. Stable Transfection

143B and SaOS-2 cells were plated at 1.6 × 10^5^ per 100 mm dish. Once confluency reached 60%, cultures were transfected with *PcDNA3.1 *or Dkk-3 using FuGENE 6 (Roche) according to the manufacturer's instructions. Once transfected, stable clones were selected with G418 (800 *μ*g/mL) starting at 48 hours after transfection and assayed for transgene expression via Western blot and real-time reverse transcription PCR (RT-PCR). Pooled transfectants were propagated and maintained in MEM-alpha with 10% FBS and 500 *μ*g/mL G418. 

### 2.4. Transient Transfection

143B and SaOS-2 cells were plated at 1.0 × 10^5^ per 100 mm dish. After reaching 80–90% confluency, both cell lines were transfected with PcDNA3.1 (Invitrogen) or Dkk-3 (Addgene) plasmids using Lipofectamine 2000 (Invitrogen) according to the manufacturer's instructions. Transfectants were maintained in MEM-alpha with 10% FBS and 1% penicillin/streptomycin for 48 h prior to cell extraction.

### 2.5. Western Blot Analysis

Cell extracts from transfected 143B and SaOS-2 cells with either PcDNA3.1 or Dkk-3 were prepared in RIPA lysis buffer containing protease inhibitors (Sigma, St. Louis, MO, USA). Cell lysates were centrifuged at 12,000 RPM for 15 minutes and the supernatant was collected. Clarified protein lysates (50 *μ*g) were electrophoretically resolved (90 minutes at 100 Volts) on denaturing 10–12% sodium dodecyl sulfate-polyacrylamide gel electrophoresis (SDS-PAGE). They were transferred to a nitrocellulose membrane (GE Healthcare, Piscataway, NJ, USA). Following blotting for 1 hour with 5% nonfat dry milk (Bio-Rad, Hercules, CA, USA) in TBST (10 mM Tris-HCL, pH 8.0, 150 nM NaCl, and 0.05% Tween-20), membranes were probed with primary antibodies and incubated overnight at 4°C. After washing twice with TBST for 5 minutes each, membranes were incubated for 1.5 hours at room temperature with secondary antibodies and visualized using the SuperSignal West Pico Chemiluminescent Substrate (Pierce). For loading control, the membrane used in initial Western blot was placed in Restore Western blotting Stripping Buffer (Thermo Scientific) for 15 minutes to remove the primary and secondary antibodies. After washing with water for 5 minutes and blocking with 5% milk for 1 hour, the membrane was probed with *β*-actin antibody (Santa Cruz Biotechnology).

### 2.6. Real-Time Reverse Transcription-Polymerase Chain Reaction (RT-PCR)

After transfecting 143B and SaOS-2 with PcDNA3.1 or Dkk-3, total RNA was isolated using the TRIzol reagent (Invitrogen, Carlsbad, CA, USA). Using a high-capacity cDNA reverse transcription kit, cDNA was synthesized from 2 *μ*g of total RNA. The sequences of the primers are as follows: Dkk-3, forward: 5′-ctgtgtgtctggggtcactg-3′; reverse: 5′-gctctagctcccaggtgatg-3′. PCR condition was as follows: 45 cycles of 30s at 95°C, 30s at 58°C, and 60s at 72°C. Relative fold change in mRNA expression compared with control was calculated using the comparative *C*_*t*_ method, where *C*_*t*_ is the cycle number at which fluorescence first exceeds the threshold.  *C*_*t*_ values were obtained by subtracting the values of *β*-actin *C*_*t*_ from the target gene *C*_*t*_. Gene-specific primer sequences are available upon request. The specificity of amplification products was verified by agarose gel electrophoresis and melting curve analysis. 

### 2.7. Immunohistochemistry Analysis of MMP-2

Paraffin-embedded osteosarcoma tissue specimens from pulmonary metastatic nodules of mice inoculated with Dkk-3-transfected 143B OS were available for immunohistochemical analysis. Four micrometer sections were deparaffinized in xylene and rehydrated in graded alcohol. Antigen retrieval was performed using 10 mM sodium citrate (pH 6.0) in a water bath at 95°C for 15 minutes. Slides were incubated with a rabbit polyclonal anti-MMP-2 antibody (Santa Cruz, CA, USA) at 1 : 300 dilution for 12 hr at RT using a humidified chamber. Slides were then incubated with a biotinylated anti-rabbit secondary antibody (Santa Cruz, CA, USA) at 1 : 200 dilution for 1 hr. Staining was visualized with diaminobenzidine using the Vectastain Elite Kit (Vector Lab, Burlingame, CA, USA) according to the manufacturer's instructions. Slides were counterstained with hematoxylin and photographed using a light microscope. 

### 2.8. Soft Agar Colony Formation Assay

Soft agar colony formation assays were performed for 143B OS cells using six-well plates. Each well contained 2 mL of 0.8% agar in complete medium as the bottom layer, 1 mL of 0.35% agar in complete medium with 6,000 cells as the feeder layer, and 1 mL complete medium as the top layer. Cultures were maintained under standard culture conditions. The number of colonies was determined with an inverted phase-contrast microscope at ×100 magnification. A group of > 10 cells was counted as a colony. 14 days after the wells were seeded, mean number of colonies from 4 independent wells was calculated.

### 2.9. Wound Healing Assay

Motility was assessed with a scratch assay to measure two-dimensional cellular movement. For *in vitro *scratch assays, transfected 143B cells (PcDNA, Dkk-3) were seeded and grown in 24-well plates at a density of 1 × 10^5^ cells/well in growth medium until they reached a confluence of ~90%. A scratch was made through each well using a sterile pipette tip. The monolayer was washed with a migration assay buffer consisting of serum-free medium plus 0.1% bovine serum albumin. The cells were monitored under the microscope (magnification ×10) for 0- and 12-hour time points after wounding. Images of cells were captured at the same position before and after incubation to assess the repair process. The experiment was repeated thrice.

### 2.10. Zymogram Assay

To determine the proenzyme and active form of MMP-2 and MMP-9, zymogram assay was done as previously described [21]. In brief, the condition medium was collected from Dkk-3-transfected 143B cells and control cells, and concentrated 20x using centricon (Millipore). Fifteen microliters of concentrated condition medium was separated by electrophoresis in 0.1% gelatin-impregnated gel (Bio-Rad). After getting renatured at room temperature for 1 hour in zymogram renature buffer, the gel was incubated overnight at 37°C in the zymogram development buffer (Bio-Rad). The gel was then stained with Coomassie Blue and destained according to the manufacturer's protocols (Bio-Rad). Gelatinolytic activity was visualized as clear bands on the gel.

### 2.11. Luciferase Reporter Assays for Wnt Inhibition

143B cells were plated in a six-well plate at a density of 1.0 × 10^5^ per well and incubated overnight. The cells were transiently cotransfected with 1.5 ug of TOPFLASH luciferase reporter plasmid (Upstate Biotechnology) and 1.5 ug of Dkk-3 (Addgene) or control PcDNA (Invitrogen) plasmids. Transfection was performed using Lipofectamine 2000 (Invitrogen) according to the manufacturer's protocol. After 48 h, cells were lysed with Glo Lysis Buffer (Promega) and luciferase activity was measured using the Bright-Glo Luciferase Assay System (Promega). 

### 2.12. *In Vivo* Tumorigenesis and Metastasis Model

4-week-old male *nu/nu *nude mice (Taconic) were housed in pathogen-free conditions. The animal protocol was approved by the Institutional Animal Care Utilization Committee. Once Dkk-3-transfected and vector-control-transfected 143B cells were grown to near confluence, they were resuspended in 0.03 mL of PBS (1 × 10^7^ cells/mL PBS) and injected percutaneously into the tibia of anesthetized nude mice. Tumor size was measured every 3 days using a caliper. The tumor volume was calculated by the formula 1/6  *π* *ab* ^2^ (*π* = 3.14; *a* is the long axis, and *b* is the short axis of the tumor). Growth curves were plotted with the mean tumor volume ± SEM from 10 animals in each group. 21 days after injection, the animals were sacrificed according to the Institutional Animal Care Utilization Committee protocol. The tumors were harvested, measured, weighed, and fixed in 10% formalin. Wet tumor weight of each animal was calculated as mean weight ± SD from 10 animals in each group. Lungs were harvested and fixed in Bouin's solution. The number of surface lung metastatic nodules was counted, and the mean number of lung nodules was compared between the two groups. Microscopic lung metastases were visualized on 5 *μ*m paraffin-embedded sections stained with H&E.

### 2.13. Statistical Analysis

Comparisons of cell viabilities between treated and control cell lines, number of colonies, fold change in levels of mRNA, and tumor weight were conducted using Student's *t*-test. For tumor growth experiments, repeated measures ANOVA was used to examine the differences in tumor volume among different time points and transfection-time interactions. Additional posttest was done to examine the differences in tumor volume between vector control and Dkk-3 transfection at each time point by conservative Bonferroni method. All statistical tests were 2 sided. Data was presented as a mean ± standard errors (SEs), and the level of significance was set at a *P* value < 0.05. 

## 3. Results

### 3.1. Transfected Dkk-3/REIC Suppresses Tumor Growth in Nude Mice and Inhibits Pulmonary Metastasis

**Figure 1:**
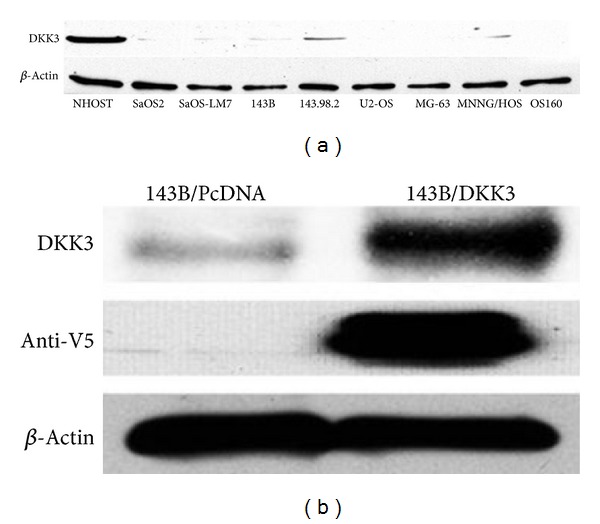
Dkk-3 protein expression of human osteoblast and OS cell lines and Dkk-3 transfected 143B cells. (a) Comparing expression of Dkk-3 in human osteoblast (NHOST) to 8 osteosarcoma cell lines (SaOS-2, SaOS-LM7, 143B, 143.98.2, US-OS, MG-63, MNNG/HOS, and OS160) via Western blot analysis using an anti-Dkk-3 antibody. All osteosarcoma cell types showed reduced expression of Dkk-3. (b) Ectopic expression of Dkk-3 in transfected 143B cells was confirmed by Western blot using an anti-V5 antibody. Dkk-3 is overexpressed in Dkk-3 transfected cells compared to PcDNA control vector.

Given the *in vitro* data supporting reduced expression of Dkk-3 in various malignant cell lines, we wanted to examine the *in vivo* effect of transfected Dkk-3 of OS cells on nude mice. 143B osteosarcoma cell line was utilized given its propensity to grow quickly and metastasize to the pulmonary fields. As seen in other cancer cell lines [11–14, 16, 22], Dkk-3 protein expression was downregulated with varying degree in all osteosarcoma cell lines (Figure 1(a)). Out of 8 OS cell lines (SaOS-2, SaOS-LM7, 143B, 143.98.2, U2-OS, MG-63, MNNG/HOS, and OS160), the 3 which showed the least expression were U2-OS, MG-63, and OS160. The human osteoblast (NHOST) in comparison showed definite greater protein expression of Dkk-3. 

**Figure 2:**
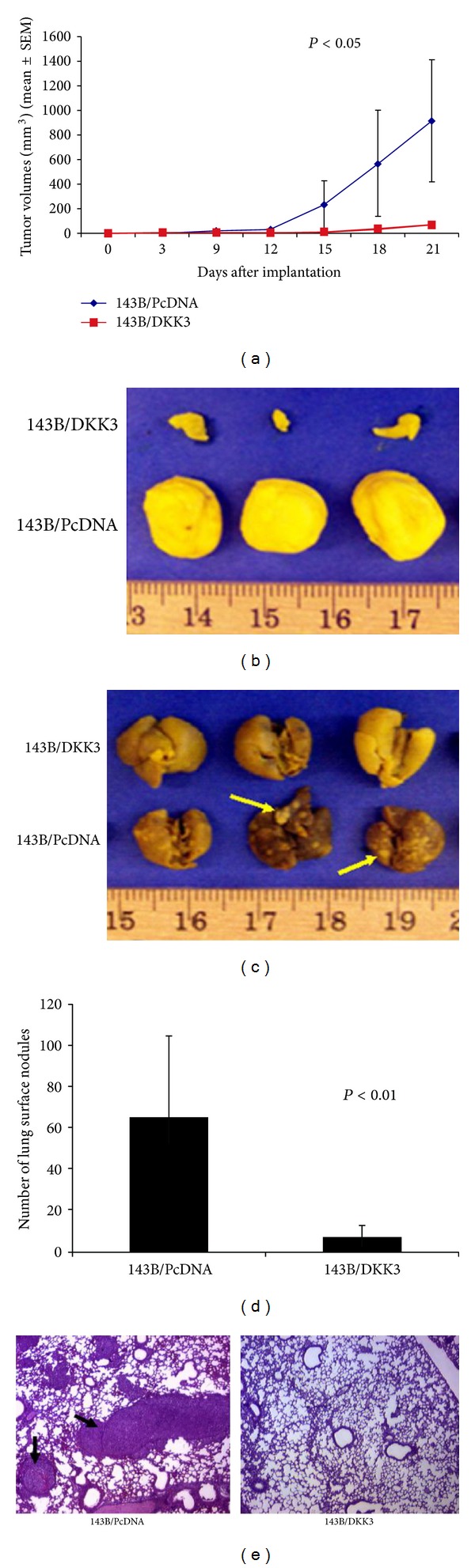
*In vivo*, Dkk-3 inhibits tumor growth in nude mice and decreases pulmonary metastasis in an orthotopic osteosarcoma mouse model. Transfected 143B cells (1 × 10^7^) with either PcDNA (control) or Dkk-3 were injected percutaneously into the tibia of anesthetized nude mice. (a) Tumor growth curve after implantation of tumor cells. Tumor size was measured every 3 days using a caliper and volume calculated (points reflect mean tumor volume), each group contained 10 mice; bars, SEM. (b) Harvested tumor tissue. Tumors were harvested 21 days after inoculation. (c) Lungs were harvested from mice injected with transfected control (PcDNA) and Dkk-3-143B OS cells. Arrows point to surface lung nodules. (d) Surface pulmonary nodules were counted under a dissecting microscope. Columns represent mean number of pulmonary surface nodules from 10 mice in each group; bars, SEM. (e) Immunohistochemical H&E staining (×40 magnification) of lung sections from mice inoculated with either transfected vector control or Dkk-3-143B OS cells. Arrows represent lung metastatic nodules.

Initially, we confirmed successful transfection of Dkk-3 into 143B cells by western blotting of the V5-tagged Dkk-3 protein using an anti-V5 antibody (Invitrogen; Figure 1(b)). Dkk-3-transfected and vector control-transfected 143B cells (equally resuspended in 0.03 mL of PBS to obtain 1 × 10^7^ cells/mL PBS) were injected percutaneously into the tibia of anesthetized nude mice and tumor size was measured every 3 days. In comparison to the control vector, the Dkk-3-transfected 143B cells showed significant slower tumor growth rate (*P* < 0.05) (Figure 2(a)). After 21 days, animals were sacrificed and tumors harvested, and Dkk-3-transfected tumors were substantially smaller compared to the control (Figure 2(b)). These results suggest that overexpression of Dkk-3 inhibits tumorigenesis. 

 Given the propensity of osteosarcoma cells to metastasize to the pulmonary fields, lungs were harvested to analyze metastatic lesions. Gross anatomy of lung fields showed significantly higher number of nodules for the vector control-transfected 143B cells compared to the Dkk-3-transfected cells (Figure 2(c)). The Dkk-3-transfected 143B cell line formed 88.7% fewer lung nodules compared to the control (Student's *t*-test; *P* ≤ 0.01) (Figure 2(d)). Histologic examination also substantiated smaller pulmonary nodules from the Dkk-3-transfected cells compared to the control-transfected cells (Figure 2(e)). These results show the remarkable inhibitory effects of Dkk-3 on pulmonary metastasis.

### 3.2. Dkk-3 Inhibits Motility, Anchorage-Independent Growth, and Cellular Viability

**Figure 3:**
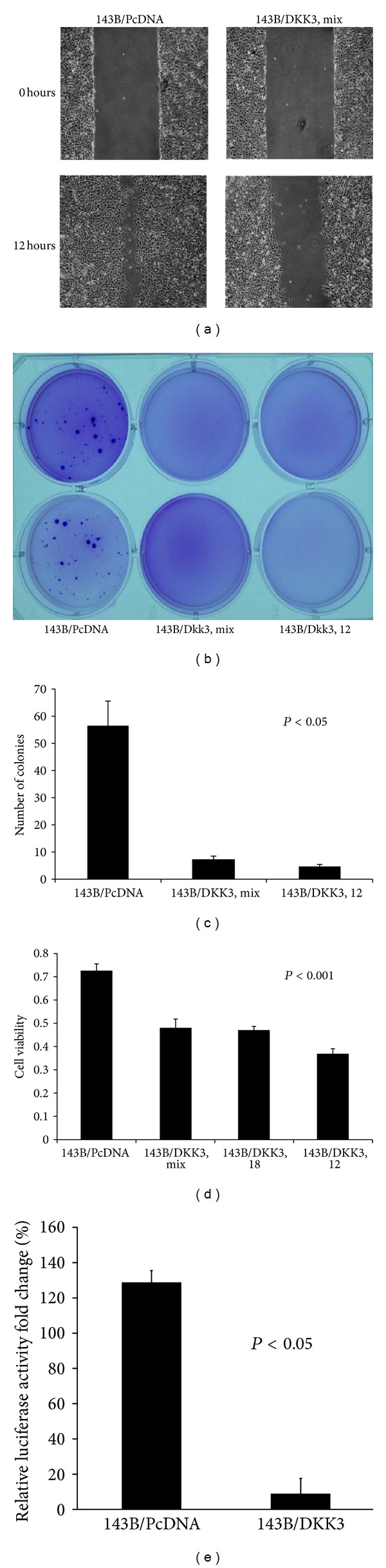
Dkk-3 inhibits cellular motility, anchorage-independent growth in soft agar and cellular viability. (a) Wound assay—photographs of scratch wounds at 0 and 12 hours after wounds were made. After 12 hours of incubation, under the fluorescence microscope, the Dkk-3-143B-transfected OS cells showed less migratory effect compared to the control vector. (b) and (c), Anchorage-independent colony formation assay showed decreased amount of colonies formed by Dkk-3-143B-transfected OS cells (both mixture of all Dkk-3 cells and a single Dkk-3 no. 12 plate) compared to transfected vector control 143B cells. (b) Photograph of soft agar colonies at 18 d after cell seeding. (c) Columns—mean number of colonies; bars—SEM. (d) MTT assay at 72 hours showed less cell viability for Dkk-3-transfected 143B cells (mix, individual cell plates nos. 12 and 18) compared to control vector. Experiments were replicated thrice. (e) Dkk-3-transfected 143B OS cells reduced TCF4 transcriptional activity compared with vector control transfection of 143B cells.

Given the results of our *in vivo *experiments, eliciting the potential molecular mechanism of Dkk-3 would be helpful in future gene targeting therapy. Cellular motility assay is an *in vitro* surrogate for assessing metastatic potential.  Figure 3(a) demonstrated the difference in cellular motility of Dkk-3-transfected 143B cells compared to the control. After 12 hours of incubation after wounding, it was clear that Dkk-3-transfected 143B cells had significant slower cellular motility compared to the control-transfected cells.

 Besides cellular motility, metastatic cancer cells also have the ability to proliferate independently of both external and internal signals that normally restrain growth. Soft agar colony formation assay was used to monitor anchorage-independent cellular growth. Figures 3(b) and 3(c) validated Dkk-3's ability to inhibit anchorage-independent growth. Dkk-3-transfected 143B cells formed 87.6% less colonies than the vector control cells (*P* < 0.05). These results confirm that Dkk-3 expression deters tumorigenesis in 143B cells.

 Along with inhibiting cellular motility and anchorage-independent growth, Dkk-3-transfected OS cells also showed a decrease cell viability compared to the control vector. MTT assay at 72 hours showed a 49.6% decrease in cell viability for Dkk-3-transfected OS cells (*P* < 0.001) (Figure 3(d)). Furthermore, to assess the inhibition of canonical Wnt activity by Dkk-3, LEF-1/TCF4 transcriptional activity was examined with TOPFLASH luciferase reporter assay for Dkk-3-transfected 143B cells and PcDNA vector control. Compared with controls, Dkk-3 reduced LEF-1/TCF4 transcriptional activity significantly (Figure 3(e), Student's *t*-test; *P* < 0.05). These results support Dkk-3 inhibits canonical Wnt activity in 143B OS cells.

### 3.3. Dkk-3 Downregulates Matrix Metalloproteinase-2 and -9 Activities (MMP-2, MMP-9)

**Figure 4:**
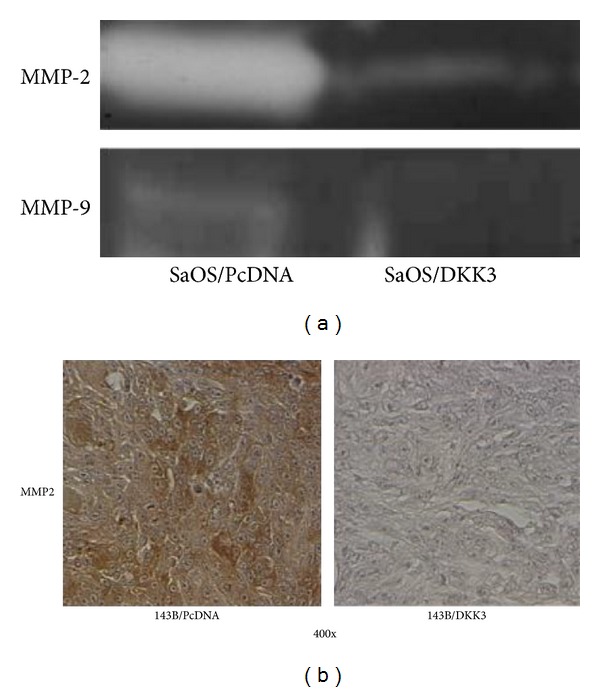
Dkk-3 deters invasive capacity of OS cells via downregulation of MMP-2 and -9 activities. (a) Gelatinolytic activities of MMP-2 and MMP-9 for Dkk-3-transfected SaOS-2 OS cells compared to vector control were evaluated by zymographic analysis. (b) Representative photographs of immunohistochemical detection of MMP-2 of pulmonary metastatic nodules in inoculated mice with Dkk-3-transfected-143B OS; magnification 400x.

We next examined the effect of Dkk-3 expression on matrix metalloproteinase (MMP) activities. Proteins of the MMP family are involved in the breakdown of extracellular matrix (ECM) in normal physiological processes as well as malignant processes, and its expression has been known to be regulated by Wnt signaling [23]. Besides ECM degradation, they also contribute significantly to tumor invasion and metastasis. Many studies have demonstrated a correlation between the overexpression of MMP with poor prognosis of various cancers, including osteosarcoma [24]. Based on these findings, we examined the effect of Dkk-3 expression on MMP-2 and MMP-9 activities. Zymography showed that ectopic expression of Dkk-3-transfected SaOS-2 cells resulted in decreased activities in both MMP-2 and MMP-9 compared to the vector control (Figure 4(a)). In addition, MMP-2 expression was also reduced by immunohistochemical analysis of pulmonary tissue from mice injected with Dkk-3/143B cells. Compared to Dkk-3 transfected tumors, pulmonary metastatic nodules from control tumors showed more intense staining of MMP-2 (Figure 4(b)).

### 3.4. Dkk-3 Inhibits the Epithelial-Mesenchymal Transition and Transcriptional Factors, Decreasing Tumorigenesis and Cellular Motility/Migration

**Figure 5:**
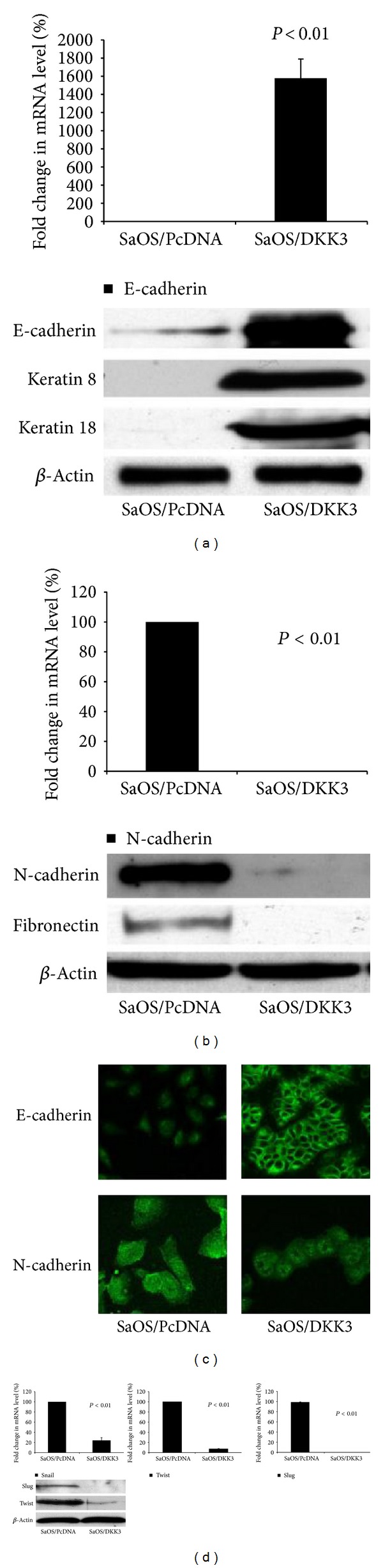
Dkk-3 inhibits epithelial-mesenchymal transitions and transcriptional factors, decreasing tumorigenesis and cellular motility/migration. (a) EMT-related marker, E-cadherin (epithelial marker) expression for Dkk-3-transfected SaOS-2 OS cells compared to control vector was determined by real-time RT-PCR. Columns, mean from 3 independent experiments; bars, SE. Western blot analysis showing expression of E-cadherin along with other epithelial markers, including Keratin 8, Keratin 18 for both subsets. Beta actin utilized as housekeeping gene. (b) EMT-related markers, N-cadherin (mesenchymal marker) fold changes in mRNA level comparing SaOS-2 OS control to Dkk-3-transfected SaOS-2 OS cells. Columns, mean from 3 independent experiments; bars, SE. Western blot analysis of mesenchymal markers (N-cadherin, fibronectin) for both subsets. (c) Immunofluorescent microscopy of N-cadherin and E-cadherin staining in transfected SaOS-2 OS cells; magnification 400x. (d) Real-time RT-PCR of transcription factors (Snail, Twist, and Slug) in transfected SaOS-2 OS cells; columns, mean from 3 independent experiments; bars, SE. Western blot analysis of Snail, Slug, and Twist in transfected SaOS-2 OS cells.

Epithelial-mesenchymal transition (EMT) is a process characterized by loss of cell adhesion and increased cell mobility. EMT may be essential for numerous developmental processes including mesoderm and neural tube formation and tumorigenesis. Cells that are involved with EMT are known to lose cell adhesion and acquire expression of mesenchymal components, which ultimately aid in cellular motility and migration. This process has been shown in various human cancers, including breast, ovarian, esophagus, gastric, colon, endometrial and synovial sarcoma [25]. E-cadherin, an essential component of cell-cell junction, is normally downregulated during EMT. In our study, overexpression of Dkk-3 led to a reversal of the EMT in SaOS-2 cells, with upregulation of epithelial markers (E-cadherin, Keratin 8, and Keratin 18) by Western blot analysis and of E-cadherin by real-time RT-PCR (1585 fold upregulation, *P* < 0.01) (Figure 5(a)). On the contrary, mesenchymal markers (N-cadherin, fibronectin) were downregulated in Dkk-3-transfected SaOS-2 cell lines. The expression of N-cadherin mRNA in Dkk-3-transfected SaOS-2 cells was markedly reduced (*P* < 0.01), and Western blot analysis revealed N-cadherin and fibronectin were greatly reduced in Dkk-3-transfected cells (Figure 5(b)). Immunofluorescent staining also suggested a greater epithelial phenotype for Dkk-3-transfected cells. Compared to control vector, Dkk-3-transfected SaOS-2 cells showed greater immunofluorescent staining for E-cadherin compared to N-cadherin (Figure 5(c)). These data further support the role of Dkk-3 in modulating EMT in OS cell lines.

 Several regulators of EMT are Wnt-responsive transcriptional repressors (Snail, Slug, and Twist) that have been shown to promote cancer progression and metastasis [25–28].  Figure 5(d) shows markedly decreased mRNA expression of Snail, Twist, and Slug in the Dkk-3-transfected SaOS-2 cells by 77.2%, 95.6%, and 99.8%, respectively (*P* < 0.01). Similarly, protein expression of these repressors is also reduced in Dkk-3-transfected cells compared to vector control cells (Figure 5(d)). This evidence suggests that in OS cells, Dkk-3 promotes a reversal of the EMT and down-regulation of transcriptional factors: Snail, Twist, and Slug.

### 3.5. Dkk-3 Downregulates S100A4, c-Met, and Phosphorylated MAPK and AKT

**Figure 6:**
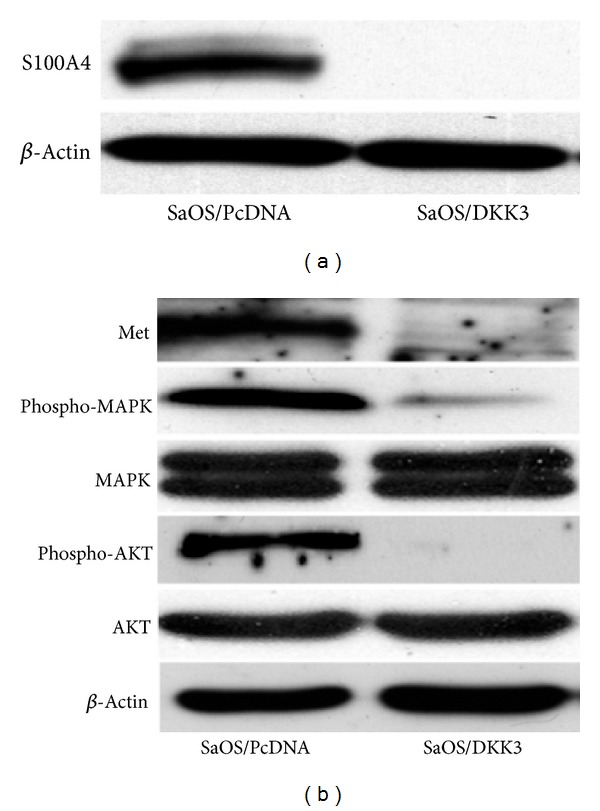
Dkk-3 modulates S100A4, Met, and downstream phosphorylated MAPK and AKT. (a) and (b) Western blot analysis demonstrating expressions of S100A4, Met, and phosphorylated/non-phosphorylated MAPK and AKT in Dkk-3 and control vector transfected SaOS-2 cells.

S100A4 is a member of the S100 family of calcium-binding proteins known to be involved in tumor metastasis [29]. S100A4 has been associated with enhanced metastatic potential, although the exact underlying mechanism is still unknown. Knockdown of S100A4 has been shown to decrease cell migration, tumorigenesis, and metastasis of OS [30, 31]. In our study, Western blot analysis showed that Dkk-3 overexpression was associated with down-regulation of S100A4 protein (Figure 6(a)), consistent with a reduction in metastatic potential. 

In addition to S100A4, c-Met and its downstream kinases (MAPK and AKT) were also examined in Dkk-3-transfected cells. Met is a known protooncogene that encodes the hepatocyte growth factor (HGF) receptor. Met activation also results in activation of MAP kinase and AKT in OS cell lines, leading to increased proliferation and motility [32]. Compared to the control vector, Dkk-3-transfected SaOS-2 cells showed decreased protein expression of c-Met and its downstream effectors phosphorylated MAPK and AKT (Figure 6(b)), further suggesting the role of Dkk-3 as an inhibitor of tumorigenesis and metastasis.

## 4. Discussion

In the present study, Dkk-3 expression was downregulated in several OS cell lines. Overexpression of Dkk-3 in OS cell line SaOS-2 led to upregulation of epithelial markers (E-cadherin, Keratin-8 and -18) and down-regulation of mesenchymal makers (N-cadherin and fibronectin), suggesting a reversal of the EMT. Furthermore, Dkk-3 expression resulted in decreased cell motility as well as reduced tumor growth and pulmonary metastasis in an orthotopic xenograft model of OS. These cellular changes are associated with reduced activities of MMP-2 and MMP-9 as well as a decrease in oncogenic c-Met, phosphorylated MAPK and AKT, and reduced expression of metastasis-associated proteins S100A4, Slug, and Twist.

The Wnt/*β*-catenin pathway has been known to play a major role in multiple cancers, including osteosarcoma. By investigating key factors which enable this pathway for tumorigenesis, the hope is to create a more targeted approach that helps to improve prognosis of OS patients. Dkk-3/REIC expression has been shown to be downregulated in multiple cancer cell lines although its exact oncogenic suppressive mechanism is still unknown. We have previously shown that human OS cell lines express several Wnt ligand and frizzled receptor combinations, suggesting an autocrine mechanism of Wnt activation in OS [33]. Furthermore, we have reported that Dkk-3 inhibits cellular invasion and motility by modulating the Wnt/*β*-catenin pathway [10]. In the present study, through *in vivo* and *in vitro* analyses, we have demonstrated that Dkk-3 has the potential capacity to inhibit tumorigenesis and metastatic properties, at least in a subset of OS tumors. Using stably transfected OS cell lines, we were able to inject these cells orthotopically into nude mice to create a clinically relevant animal model of OS and to examine both local tumor growth as well as pulmonary metastasis from the primary site. Consistent with our *in vitro* experiments, Dkk-3 demonstrates a remarkable suppressive effect on tumor growth. Furthermore, we observed pulmonary metastatic nodules that were present in much lower levels in mice injected with Dkk-3 transfected cells. These findings strongly suggest that targeting Dkk-3 for antitumorigenic and antimetastatic purposes should be investigated further. 

S100A4 and c-Met have been shown to be a key prognostic marker in multiple cancers. *In vivo* experiments have shown evidence of S100A4 direct involvement in tumor progression and metastasis [29]. In our study, down-regulation of S100A4 in Dkk-3-transfected OS cells is consistent with the observation that Dkk-3 can suppress cellular invasion and motility. Not only is oncogenic c-Met expression decreased in Dkk-3-transfected OS cells, but its downstream activation of MAPK and AKT is similarly downregulated. These results show a strong correlation between Dkk-3 and antitumor and antimetastatic effects.

## 5. Conclusions

Despite current therapies using intensive neoadjuvant/adjuvant chemotherapy and wide surgical resection, we still have not made a significant impact in the prognosis of metastatic osteosarcoma. Multiple canonical and noncanonical Wnt/*β*-catenin pathway inhibitors and agonists are being explored for potential gene targeting therapies. Our results, both *in vitro* and *in vivo*, are intriguing and certainly suggest an important role for Dkk-3 in the pathobiology of human OS. The mechanisms of action of Dkk-3 are likely to involve multiple important oncogenic pathways and processes (i.e., S100A4, Met, MMPs, and EMT) and their complex interactions. Reexpressing Dkk-3 in Dkk-3-deficient OS can potentially prove to be of benefit as a preventive or therapeutic strategy.
